# Chemical Composition and Biological Activities of the Nord-West Romanian Wild Bilberry (*Vaccinium myrtillus* L.) and Lingonberry (*Vaccinium vitis-idaea* L.) Leaves

**DOI:** 10.3390/antiox9060495

**Published:** 2020-06-05

**Authors:** Bianca-Eugenia Ștefănescu, Lavinia Florina Călinoiu, Floricuța Ranga, Florinela Fetea, Andrei Mocan, Dan Cristian Vodnar, Gianina Crișan

**Affiliations:** 1 Department of Pharmaceutical Botany, “Iuliu Hațieganu” University of Medicine and Pharmacy, 23, Ghe. Marinescu Street, 400337 Cluj-Napoca, Romania; stefanescu.bianca@umfcluj.ro (B.-E.Ș.); mocan.andrei@umfcluj.ro (A.M.); gcrisan@umfcluj.ro (G.C.); 2 Institute of Life Sciences, University of Agricultural Sciences and Veterinary Medicine Cluj-Napoca, Calea Mănăştur 3-5, 400372 Cluj-Napoca, Romania; 3 Faculty of Food Science and Technology, University of Agricultural Sciences and Veterinary Medicine Cluj-Napoca, Calea Mănăştur 3-5, 400372 Cluj-Napoca, Romania; florcutza_ro@yahoo.com (F.R.); florinelafetea@yahoo.com (F.F.); 4 Laboratory of Chromatography, Institute of Advanced Horticulture Research of Transylvania, University of Agricultural Sciences and Veterinary Medicine, 400372 Cluj-Napoca, Romania

**Keywords:** bilberry, lingonberry, polyphenols, antioxidant compounds, antimicrobial activity, antimutagenicity, altitude variations

## Abstract

This study was performed to evaluate and compare the in vitro antioxidant, antimicrobial, and antimutagenic activities, and the polyphenolic content of the Nord-West Romanian wild bilberry (*Vaccinium myrtillus* L.) and lingonberry (*Vaccinium vitis-idaea* L.) leaves from three different natural habitats (Smida, Turda, Borsa). In the case of both species, the flavanols level was higher in Smida habitat (altitude 1100 m), whereas quercetin derivates were more abundant in Borsa habitat (altitude 850 m). The bilberry leaf extracts contained in the highest amounts the feruloylquinic acid (59.65 ± 0.44 mg/g for Borsa habitat) and rutin (49.83 ± 0.63 mg/g for Borsa habitat), and showed relevant 2,2-diphenyl-1-picrylhydrazyl (DPPH) antioxidant activity (271.65 mM Trolox/100 g plant material for Borsa habitat, 262.77 mM Trolox/100 g plant material for Smida habitat, and 320.83 mM Trolox/100 g plant material for Turda habitat), for all the three extracts. Gallocatechin was the dominant flavanol in lingonberry species, with the highest amount being registered for Smida habitat (46.81 ± 0.3 mg/g), revealing a DPPH antioxidant activity of 251.49 mM Trolox/100 g plant material. The results obtained in the antimicrobial tests showed that the best inhibitory effect among bilberry species was attributed to the Turda (altitude 436 m) and Smida locations, against both Gram-positive and Gram-negative bacterial strains. For lingonberry, the differences in habitat did not influence the antibacterial effect, but the antifungal effect, only in the case of *Candida zeylanoides*. A strong antimutagenic effect was registered by the bilberry leaves toward *Salmonella typhimurium* TA100. Our study may be able to provide a better understanding of the correlation between natural habitat conditions and the accumulation of secondary metabolites and their related bioactivities in studied leaves.

## 1. Introduction

Most recent epidemiological studies have reported that certain medicinal plants can be responsible for preventing the development or evolution of several diseases [1,2,3,4]. The naturally-derived antioxidants are a topic of major interest considering their proven health effects on humans [2,5], but also to gradually replace the synthetic antioxidants that have been reported as endocrine disrupters or even carcinogenic compounds [6,7]. Dietary polyphenols have diverse therapeutic uses and several proven biological properties [1,8,9,10,11], being of important consideration to study their varieties in medicinal plants and natural foods [12].

The development of newly plant-derived functional products and nutraceuticals, known as edible sources with high antioxidant content, have been the intensively studied research topics in recent years [13]. Among them, *Vaccinium* species are constantly reported for their diversity in phenolic compounds [14,15,16], whereas cranberry (*Vaccinium macrocarpon* Ait.) and bilberry (*Vaccinium myrtillus* L.), being more debated than lingonberry (*Vaccinium vitis-idaea* L.), contributed to their high consumption rate under several forms: as fresh fruits, processed products, and dietary supplements. Recent literature reported that lingonberry occupies a significant position in the antioxidant and antimicrobial capacity ranking of *Vaccinium*-derived species [17,18].

Bilberry (*Vaccinium myrtillus* L.), also known as the European blueberry, and lingonberry (*Vaccinium vitis-idaea* L.), commonly known as cowberry or partridgeberry, are two small, spontaneous growing shrubs belonging to the genus *Vaccinium*, *Ericaceae* family. Their berries mature from July to September, while the ripeness time is highly affected by the site conditions, precisely altitude, and habitat type. Usually, higher altitudes generate later plant ripening when compared with lower elevations.

The bilberry and lingonberry leaves are the main by-products of berry harvesting and recent investigations [14,19] have reported a significantly higher content of phenolic compounds in the leaves and stems of *Vaccinium* species in contrast to the berries, in line with the strongest antioxidant activities registered by these aerial parts than fruits [20], indicating that they may be utilized as an alternative source of bioactive natural products for the development of food supplement, nutraceuticals, or functional food. Literature studies have shown that the leaves of bilberry and lingonberry contain fewer anthocyanins than fruits, but the content of phenolic compounds is higher in leaves than in fruits [16,21,22]. Several studies have reported the presence of hydroxycinnamic acids, flavonols, proanthocyanidins, cinchonains, and iridoids in the bilberry leaves [19,23,24,25]. Traditionally, bilberry leaves extracts are used for treating urinary tract affection and diabetes. Owing to the presence of various phenolic compounds, bilberry leaves also have antibacterial, anti-inflammatory, and antioxidant activities [26,27,28]. Chemical composition and biological properties of lingonberry leaves are similar to those of bilberry. Phenolic compounds found in lingonberry leaves are hydroxycinnamic acids, proanthocyanidins. flavonols, cinchonains, iridoids, and arbutin derivatives [14,23,27]. Extracts of lingonberry leaves have shown multiple beneficial diuretics and antiseptic properties for the urinary tract, anti-cough, phlegm removing, anti-inflammatory, neuroprotective, and antioxidant activity [21,29,30].

The genetic factor must be considered when referring to polyphenol biosynthesis in the different parts of the plant, including leaves. Moreover, the biotic and abiotic conditions may be responsible for certain variations (increases or decreases) in phenolic concentration, as reported in the recent literature for bilberry leaf and stem [19,24] and lingonberry leaf [25,31]. A multitude of environmental factors change with the altitude of the growing site, precisely precipitation, mean temperature, soil, wind speed, low- and high-temperature extremes, duration of snow cover, length of vegetation period, and intensity of radiation under clear sky conditions. Enhanced UV-B radiation and lower temperatures at high altitudes have been constantly debated as having an impact on plant secondary metabolism [32,33]. As a protective mechanism towards damage induced by excessive UV-B radiation, plants support and stimulate the biosynthesis of UV-B-absorbing phenolic compounds with an antioxidant capacity [32,34]. The stimulation of enzymes responsible for flavonoid biosynthesis in UV-enhanced radiation experiments was highly underlined [35,36].

The latitude-related factor was discussed in particular for *V. myrtillus* L., being reported for the high influence on the quality and quantity of phenolic compounds [24,37,38,39,40], suggesting that higher phenolic amounts may be supported by northern latitudes, altitude, and sunny weather. However, most studies have aimed to investigate an individual morphological part of the bilberry plant, with fruits as most debated, and leaves in a small percentage. In this context, this study aims to provide a better understanding of the correlation between natural habitats and the accumulation of phenolic compounds in the leaves of *Vaccinium myrtillus* L. and *Vaccinium vitis-idaea* L. and their related bioactivities: antioxidant, antimicrobial, and antimutagenic. Thus, the investigation on the differences, derived from natural habitats within the same region (Nord-West), on polyphenolic content of the Romanian wild bilberry and lingonberry leaf extracts could be useful to broaden the knowledge on this field.

Considering that the chemical composition of the Nord-West Romanian wild bilberry and lingonberry leaves has never been the subject of a scientific paper to best of our knowledge, this study aimed to determine the phenolic composition of bilberry and lingonberry leaves and to measure their antioxidant, antibacterial, antifungal, and antimutagenic activities, whereas the antimutagenic and antimicrobial activities of the leaves are of significant novelty. Furthermore, the differences between the three different natural habitats of Romanian bilberry and lingonberry leaves were also investigated.

## 2. Materials and Methods

### 2.1. Plant Samples and Growing Conditions

The leaves of bilberry (*Vaccinium myrtillus* L.) and lingonberry (*Vaccinium vitis-idaea* L.) were collected in the autumn (September) of 2017 from spontaneous species of three different locations in Romania, differing in altitude and habitat type: (1) Turda (46°32′00″ N, 23°52′00″ E), Cluj County; (2) Smida (46°38′33″ N, 22°52′49″ E), Cluj County; and (3) Borsa (47°39′19″ N, 24°39′47″ E), Maramures County. Leaves of both species were randomly sampled from ca. 10 shrubs in the same 20 m × 20 m area for each habitat. The plant material was dried at room temperature for 7–10 days and grounded to a fine powder and kept in the dark prior to analyses. The results were calculated based on the dried and grounded plant material/powder. The numbers of Plant Voucher Specimens are VM103 and VVI105.

### 2.2. Description of Habitats

Turda is a municipality in the county of Cluj, Transylvania, Romania, and it is located about 30 km southeast of Cluj-Napoca. Turda developed mainly on the left side of the Arieș river. The minimum altitude is 310 m in the eastern extremity, on the Arieș valley, and the maximum is in the northeast of the city, on Slăninii Hill (436 m), from where the leaves were collected. The karst relief is present and develops into soluble rocks (limestone, salt, gypsum), being characterized by mineral soils. The climate in September is quite dry with 44 mm of rainfall, and involves a maximum temperature of 23 °C and a minimum of 15 °C. The collection place had a moderate solar exposition considering the slope exposure [41].

Smida is located in the heart of the Apuseni Natural Park, a protected area that is among the last large areas of large, forested karst (spreading its wild beauty on approximately 76,000 hectares) throughout Europe. Smida village is at an altitude of 1100 m and benefits from a moderate continental climate, whereas in September, there is a maximum temperature of 23 °C and a minimum of 1 °C, with 6.5 mm of rainfall. The soil is characterized by acid brown soils with medium texture, and good aquatic drainage considering the winters rich in snow. It possesses large areas of natural forests and meadows, with a variety of fauna and flora. The relief is a karstic one, well developed, and made up of caves [42]. The collection place had partial sun exposure considering the open-spaced areas surrounded by forest.

The Borsa town is located in the south of Maramureș county, Transylvania, Romania at an altitude of 850 m in the Rodnei Mountains, on the Vișeu river valley. The relief of the area is mountainous, very rugged, and with steep slopes and high-level differences, being characterized by the moderate continental climate sector, with a maximum temperature of 13 °C and a minimum of 8 °C, and with the average annual rainfall of 1100 mm and permanent exposure to the advection of the western air masses of oceanic nature, whose characteristics are reflected in the evolution of all climatic elements. The collection place is characterized by acid brown soils and good solar exposition [43].

### 2.3. Chemicals and Reagents

Catechin, chlorogenic acid, quercetin, cyanidin chloride, and gallic acid used as standards for the HPLC-DAD-ESI-MS analysis were purchased from Sigma-Aldrich (Steinheim, Germany). Folin–Ciocalteu’s phenol reagent, sodium carbonate (Na2CO3), sodium nitrate (NaNO2), hydrochloric acid (HCl), aluminum chloride (AlCl3), sodium hydroxide (NaOH), acetic acid, acetonitrile, methanol, ethanol, DPPH (2,2-diphenyl-1-picrylhydrazyl), and Trolox (6-hydroxy-2,5,7,8-tetramethylchroman-2-carboxylic acid) were purchased from Sigma-Aldrich (Steinheim, Germany). For antimicrobial assays, Mueller–Hinton agar, thioglycollate broth with resazurin, and Mueller–Hinton broth were purchased from BioMerieux (France), and Tween 80 and Broth Malt medium were purchased from Sigma-Aldrich (Steinheim, Germany).

### 2.4. Ultrasound-Assisted Extraction Procedure

The fine powder obtained from the leaves (0.25 g) was extracted with 7 mL 40% *v*/*v* ethanol in water for 30 min in an ultrasonic bath, at 20 °C. After centrifugation (5000 rpm for 10 min at 24 °C), the supernatant was filtered and stored (−18 °C) until analysis (total phenolic content, total flavonoid content, total anthocyanin content, antioxidant, antimutagenic and antimicrobial activities, and HPLC-DAD-MS analysis).

### 2.5. Analysis of Phenolic Compounds

#### 2.5.1. HPLC-DAD-ESI-MS Analysis

Identification and quantification of phenolic compounds in the leave extract were performed on an HPLC-DAD-ESI-MS system consisting of an Agilent 1200 HPLC with DAD detector, coupled to an MS-detector single-quadrupole Agilent 6110. For phenolic compounds’ separation, the Eclipse column, XDB C18 (4.6 × 150 mm, particle size 5 µm) (Agilent Technologies, USA), was used at 25 °C. The binary gradient was prepared from 0.1% acetic acid/acetonitrile (99:1) in distilled water (*v*/*v*) (solvent A) and 0.1% acetic acid in acetonitrile (*v*/*v*) (solvent B) with a flow rate of 0.5 mL/min, according to the elution program described by Dulf et al. [44]. For MS fragmentation, the ESI (+) module was used, with a scanning range between 100 and 1200 m/z, capillary voltage 3000 V, at 350 °C, and with a nitrogen flow of 8 l/min. The eluent was monitored by DAD, and the absorbance spectra (200–600 nm) were measured and collected during each run. For analyzing the data, Agilent ChemStation Software (Rev B.04.02 SP1, Palo Alto, CA, USA) was performed. The phenolic compounds from the extracts were identified by comparing the retention times, UV visible, and mass spectra of the peaks with four reference standards, as follows: the compounds of the flavanol subclass were quantified using the calibration curve performed with catechin standard on the concentration ranges of 10–200 µg/mL and expressed as equivalents of catechin (mg catechin/g plant material) (*r*^2^ = 0.9985); for the hydroxycinnamic acid subclass, the compounds were quantified using the calibration curve performed with chlorogenic acid on the concentration range of 10–50 µg/mL, expressed as chlorogenic equivalents (mg chlorogenic acid/g plant material) (*r*^2^ = 0.9937); flavonols were quantified using the calibration curve performed with quercetin on the concentration ranges of 10–200 µg/mL, expressed as quercetin equivalents (mg quercetin/g plant material) (*r*^2^ = 0.9951); and anthocyanins were quantified using the calibration curve made with cyanidin on the concentration ranges of 10–100 µg/mL, expressed as cyaniding equivalents (mg cyanidin/g plant material) (*r*^2^ = 0.9951).

#### 2.5.2. Total Phenolic Content

The determination of total phenolic content (TPC) was performed by the Folin–Ciocalteu method [44,45]. Briefly, 25 µL of sample extract was combined with 125 µL of Folin–Ciocalteu reagent (0.2 N) and 100 µL of sodium carbonate solution (Na_2_CO_3_, 7.5% *w*/*v*). Afterward, the mixture was incubated for 2 h in the dark at room temperature (25 °C). The absorbance was recorded at 760 nm, using ethanol as blank. A standard curve was prepared using gallic acid (0.01–1 mg/mL), and the TPC in the extract was expressed as gallic acid equivalents (GAE) (mg GAE/100 g plant material).

#### 2.5.3. Total Flavonoid Content

Total flavonoid content (TFC) was determined by a spectrophotometric method [46] based on the formation of a complex flavonoid—aluminum. Shortly, 1 mL of sample extract was mixed with 0.3 mL NaNO_2_ (5%); after 5 min, 0.3 mL AlCl_3_ (10%) was added; afterward, 2 mL NaOH (1M) and water to a total volume of 10 mL. The absorbance was measured immediately, at 510 nm. A standard curve was prepared using quercetin (0.117–1 mg/mL) and the TFC was expressed as quercetin equivalents (QE) (mg QE/ 100 g plant material).

#### 2.5.4. Total Anthocyanin Content

The total anthocyanin content (TAC) was determined by UV/visible spectrophotometric method [47]. The extracts were diluted with 40% ethanol, and the absorption was measured at 530 nm using a Jasco UV-VIS Spectrophotometer (V-530 double beam, Tokyo, Japan). The anthocyanin content was estimated as cyanidin-3-glucoside at 530 nm using a molar absorptivity coefficient of 26,900 and was expressed as milligrams per 100 g of plant material [47].

### 2.6. DPPH Free-Radical-Scavenging-Assay

DPPH free-radical–scavenging activity was performed using the method described by Ebrahimabadi et al. [48] with slight modifications. First, 250 µL of each leaf hydroethanolic extract was mixed with 1750 µL of freshly prepared DPPH solution (0.1 mM in 40% ethanol). The absorbance was measured after 30 min of rest under dark conditions, at 517 nm, using the spectrophotometer Biotek and 40% ethanol as blank. In the DPPH assay, the antioxidant activity of the extracts was evaluated using the calibration curve performed with Trolox, and then the absorbance was recorded for all the tested extracts, to calculate the percentage inhibition (expressed as percentage inhibition of the DPPH radicals). The percentage inhibition (I%) was calculated as I% =[ (A_B_ − A_A_)/ A_B_] × 100, where A_B_ = absorbance of blank and A_A-_ = absorbance of hydroethanolic extract.

### 2.7. Antimicrobial and Antifungal Capacity

#### 2.7.1. Stains and Cultivation Conditions

To determine the antimicrobial activity for all extracts, six bacterial strains were used: three Gram-positive bacteria: *Staphylococcus aureus* (ATCC 49444), *Enterococcus faecalis* (ATCC 29212), *Rhodococcus equi* (ATCC 6939), and three Gram-negative bacteria: *Pseudomonas aeruginosa* (ATCC 27853), *Klebsiella pneumonia* (DSMZ 2026)*, Escherichia coli enterotoxigen* (ATCC 25922). All tested microorganisms were obtained from the Food Biotechnology Laboratory, UASVM CN, Romania.

#### 2.7.2. Microdilution Technique

Evaluation of the antimicrobial activity was done according to the guidelines of the Clinical Laboratory Standards Institute (CLSI) [49], using the standard broth microdilution technique for bacteria that grow aerobically, with slight modifications. Briefly, all the bacteria were cultured on Mueller–Hinton agar, followed by their storage at 4 °C and subculture once a month. Before antibacterial susceptibility testing, each strain was inoculated on Mueller–Hinton agar plates and incubated at 37 °C for 24 h. The medium used for susceptibility testing was Mueller–Hinton broth. Inoculums (density of 0.5 in McFarland scale) were prepared in a 0.9% NaCl sterile solution. Then, tested strains were suspended in Mueller–Hinton broth medium, to give a final density of 2 × 10^5^ colony-forming units (CFU)/mL. The inoculum was stored at 4 °C for further use. Determinations of minimum inhibitory concentrations (MICs) were performed by a serial dilution technique using 96-well plates. The 100 µL Mueller–Hinton broth was placed into each of the 96 wells of the microplates. Aliquots of 100 µL of each extract (concentration of 0.1 g/mL) were added into the first rows of the microplates and twofold dilutions of the extracts were made by dispensing the solutions into the remaining wells. Then, 10 µL of the culture suspensions was inoculated into the wells. We used ethanol (40%) in water as a control. The microplates were incubated for 24–48 h at 37 °C. The MIC of the plant extracts was detected after the addition of 20 μL (0.2 mg/mL) of resazurin solution to each well, and the plates were incubated for 2 h at 37 °C. A change from blue to pink indicates the reduction of resazurin and, therefore, bacterial growth. The MIC was defined as the lowest concentration of the extract that inhibited the growth of the bacterial strain [50], which respectively prevented this color change. The minimum bactericidal concentrations (MBCs) were determined by serial subcultivation of 2 μL into 96-well plates containing 100 μL of Mueller–Hinton broth per well and further incubation for 48 h at 37 °C. The MBC was defined as the lowest concentration of the tested extract/compound/antibiotic killing the majority (99.9%) of bacterial inoculum, thus with no visible growth [50]. Streptomycin (Sigma P 7794, Santa Clara, CA, USA) (0.05–3 mg/mL) was used as a positive control for bacterial growth. Water was used as a negative control.

#### 2.7.3. Antifungal Assay

Evaluation of the antifungal activity was done according to the guidelines of the CLSI [51], using the reference method for broth dilution antifungal susceptibility testing of yeasts, with slight modifications. To determine the minimum inhibitory concentration (MIC) and minimum fungicidal concentration (MFC) of the tested extracts, three fungi were used: *Candida albicans (ATCC 10231), Candida zeylanoides (ATCC 20367*), and *Candida parapsilosis (ATCC 22019)*. All the tested fungal strains were obtained from the above-mentioned source. The cultures were stored on malt agar at 4 °C and subcultured monthly. Before antifungal susceptibility testing, each strain was inoculated on malt agar plates to ensure optical growth characteristics and purity. The medium used for susceptibility testing was broth malt. The initial density of *Candida* spp. was approximately 2 × 10^6^ colony-forming units/mL (CFU/mL). Inoculums (density of 0.5 in McFarland scale) were prepared in a 0.9% NaCl sterile solution. Then, tested strains were suspended in broth malt medium, to give a final density of 1.5 × 10^5^ CFU/mL. For the minimum inhibitory concentration test, the broth microdilution method was applied by preparing a serial of dilutions in 96-well plates. The 100 µL medium was placed into each of the 96 wells of the microplates. Aliquots of 100 µL of each extract diluted in 0.85% saline (concentration of 0.1 g/mL) were added into the first rows of the microplates, and twofold serial dilutions were made by dispensing the solutions into the remaining wells. Then, 10 µL of the inoculum was added to the wells. Plates were incubated at 28 °C for 72 h on a rotary shaker. Minimum inhibitory concentration (MIC) values were determined by adding resazurin (20 μL, 0.02%) followed by incubation for 2 h. The MIC was defined as the lowest concentration required to inhibit the growth of the fungal strain (observed through a binocular microscope). The MFCs were determined by serial subcultivation of 2 μL of tested extracts dissolved in medium and inoculated for 72 h into microtiter plates containing 100 μL of broth per well, followed by further incubation 72 h at 28 °C. The lowest concentration with no visible growth was defined as the MFC, indicating the death of 99.9% of the original inoculum. The positive control used was fungicide fluconazole (1–3500 µg/mL) (Sigma F 8929, Santa Clara, CA, USA), while the negative control used was water. All the tests were done in duplicate and repeated thrice.

### 2.8. Mutagenic and Antimutagenic Assay

According to the plate incorporation method [52], described in more detail by Sarac and Sen [53], the plant extracts were tested for mutagenicity and antimutagenicity towards *S. typhimurium* TA98 and *S. typhimurium* TA100, whereas the positive controls used were 4-nitro-ophenylenediamine (4-NPD, 3 mg/plate) for TA98 and sodium azide (NaN3, 8 mg/plate) for TA100. The negative control was ethanol/water (1:1, *v*/*v*), and the concentration of plant extracts was established to 5 mg/plate. According to the equation described by Ong et al. [54], the antimutagenicity was calculated as follows: %Inhibition = [1 − T/M] × 100, where T is the number of revertants per plate in the presence of mutagen and the plant extract, and M is the number of revertants per plate without plant extract (positive control). The antimutagenicity of the reference mutagens in the absence of the plant extract was defined as 0% inhibition. For each of the two species, the testing was done in duplicate with three subsamples each, and in accordance, the data are reported as the mean ± standard deviation (SD). The following percentage ranges were used to express the antimutagenicity: strong: 40% or more inhibition; moderate: 25–40% inhibition; low/none: 25% or less inhibition [55].

### 2.9. Statistical Analysis

All of the analyses were done in triplicate, and the data were reported as the means ± standard deviation (SD). The statistical differences among the leave extracts of the three different locations for each type of species were performed using one-way analysis of variance (ANOVA) (Tukey multiple comparison tests) via GraphPad Prism Version 8.0.1 (Graph Pad Software Inc., San Diego, CA, USA). Differences between means at the 5% level were reported to be statistically significant.

## 3. Results and Discussion

### 3.1. Phenolic Profile of Wild Bilberry and Lingonberry Leaves

In this study, in the leaves of bilberry and lingonberry, 21 phenolic compounds were identified, originating from four phenolic groups: hydroxycinnamic acids, flavonols, flavanols, and anthocyanins, whereas 19 were found in bilberry leave extracts and 18 in lingonberry leave extracts (Table 1). In the case of bilberry leaves, the most abundant compounds for all three locations were represented by the flavonols class comprising only quercetin derivates. The second most abundant class was flavanols. For lingonberry leaves, the most abundant class of compounds was flavanols, as reported in the literature [56], for all the three different altitude habitats, followed by hydroxycinnamic acids. In the case of lingonberry leaves, the flavonols class registered small levels for each compound, except rutin. Moreover, the anthocyanins group was not detected.

The flavanols identified among the two studied species were catechin, epicatechin, gallocatechin, epigallocatechin, two procyanidin dimers, and procyanidin trimer. The procyanidin dimers and trimers are known as proanthocyanidins as well.

In the case of bilberry leaves, for almost all flavanols, the *V. myrtillus* leaves from Smida (VMS) reported the highest amounts. Exceptions were catechin and procyanidin dimers II, in which case *V. myrtillus* leaves from Turda (VMT) presented significantly higher values. The major flavanol identified was procyanidin trimer in all three natural habitats, whereas the VMS had the highest value (24.30 ± 0.72 mg/g plant material), closely followed by VMT, while *V. myrtillus* leaves from Borsa (VMB) registered a 2.5-fold lower value. In particular, gallocatechin was twofold more in VMS than VMT and threefold more when compared with VMB. Epigallocatechin presence only in VMS contributes to the range of differences found among the three different locations. Procyanidin dimer I was not detected in the bilberry leaves of any of the three habitats, whereas epicatechin was the minor compound identified. The VMB leaves had the lowest values among all the flavanols identified, whereas epigallocatechin, epicatechin, and procyanidin dimer I were not present. Compounds present in lower proportions were catechins, in line with the previous results on bilberry leaves from Northern Europe [24]. Significant differences in gallocatechin, epigallocatechin, epicatechin, and procyanidin trimer were detected among locations, and up to threefold higher levels (in the case of gallocatechin) were measured, which can be linked to specific growth conditions of the sites (soil, solar exposure, microclimatic conditions). The habitat can specifically influence the amounts of phenolics as follows: either by the influence of pedological or climatic factors and their interactions [57]. Likewise, Martz et al. [24] reported that high-light-intensity location, higher altitudes, and/or latitudes contributed to more than twofold higher levels of phenolics in the leaves in contrast to lower altitudes or low-light-intensity sites.

Exceptionally high levels of flavanols were quantified in lingonberry leaves, in agreement with the results of previous studies [14,16,56,58], whereas the most recent study of Tian et al. [59] found, as the two most common flavanols, (+)-catechin and (-)- epicatechin, at the highest level in lingonberry (*V. vitis-idaea*) leaf extract (118 mg/100 mL). In our study, gallocatechin was quantified in high amounts, whereas epigallocatechin was detected in significant levels ranging from 23.35 ± 0.61 to 35.97 ± 0.23 depending on the habitat. Their occurrence has never been reported in lingonberry so far, only in bilberry leaves [23] and bilberry stems [19]. However, Bujor et al. [14] reported only a trace amount in lingonberry leaves and quantified the gallocatechin in lingonberry stems. In our study, *V. vitis-idaea* leaves from Smida (VVIS) registered the highest values among almost all the flavanols identified, except for procyanidin trimer and procyanidin dimer II. Gallocatechin was the major flavanol identified in all the three locations, whereas VVIS had the highest amount (46.81 ± 0.38 mg/g plant material), while epigallocatechin and catechin were close behind. Epicatechin was the minor compound identified, and only in *V. vitis-idaea* leaves from Borsa (VVIB); moreover, VVIB registered the highest values for procyanidin dimer II and procyanidin trimer. Furthermore, recent literature reported rich contents of procyanidin dimers and trimers in the extracts of lingonberry leaf (85 mg/100 mL) [59]. All these results underline that rising concentrations of flavanols and especially gallocatechin, epigallocatechin, and catechin in lingonberry leaves, as well as procyanidin trimer in bilberry leaves, were observed in the habitats with higher altitude. This fact was explained in the previously reported results, whereas an increase of flavonoids level with elevation in herbal plants [60] and bilberry leaves [24] was registered. According to the literature, catechin dominated in the red berries, lingonberry, and cranberry, while epicatechin dominated in blue and blackberries [56], in agreement with our findings.

*V. vitis-idaea* L. yielded greater amounts of gallocatechin, epigallocatechin, catechin, and procyanidin dimer I with increasing altitude and its related climatic and soil conditions, except lower levels were found at the altitudes of 1100 when compared with 850 m in the case of procyanidin dimer II and procyanidin trimer (Table 1). The variation of the flavonoid fraction turned out to be closely related to the altitude-derived conditions, because we found the percentages of four out of six flavonoid compounds rising significantly at the highest altitude. It can be concluded that environmental factors at higher altitudes lead to elevated levels of flavanols, with gallocatechin and epigallocatechin above all, in dried and grounded lingonberry leaves.

Hydroxycinnamic acids are the most widespread phenolic acids in plants, which are described as cinnamic acid-derived compounds. Four derivates of hydroxycinnamic acid were identified: chlorogenic acid, feruloylquinic acid, dicaffeoylquinic acid, and caffeoylarbutin. In the case of bilberry species, VMB together with its environmental-derived conditions (good solar exposure, acid brown soil, low-temperature range) presented the highest levels among the four compounds, except for caffeoylarbutin and dicaffeoylquinic acid, which were not detected. The major compound reported was feruloylquinic acid (59.65 ± 0.44 mg/g plant material in VMB), followed by chlorogenic acid (5.94 ± 0.05 mg/g plant material in VMB) as 10-fold less than feruloylquinic acid. The study of Martz et al. [24] indicated that bilberry leaves from higher latitudes and higher altitudes (boreal forests in Finland, thus low solar exposure) had lower levels of chlorogenic acid derivatives. In the recent paper investigating the Finnish bilberry (*V. myrtillus* L.) leaf extract, hydroxycinnamic acid derivatives represented 82% of the total content of phenolics, mostly as 3-O-caffeoylquinic acid, whereas other hydroxycinnamic acids (coumaric acid, caffeic acid, and ferulic acid) were identified as esters of acids or hexoses [59].

Concerning the lingonberry species, a specific hydroxycinnamic acid was found, precisely the caffeoylarbutin (not detected in bilberry leaves), with an increase of three- to fourfold when compared with dicaffeoylquinic acid, depending on the location. Similarly, Liu et al. [25], Tian et al. [16], and Hokkanen et al. [23] found 2-caffeoylarbutin as the major caffeic acid derivative. The lowest levels of all hydroxycinnamic acids were reported for VVIS, whereas the highest levels were reported for VVIB (caffeoylarbutin). The major compound was again feruloylquinic acid (33.42 ± 0.37 mg/g plant material), as almost half the amount when compared with bilberry species.

Bidel et al. [61] found that the amount of hydroxycinnamic acid highly increased with a higher photosynthetic active radiation (PAR) level, while Li et al. [62] also reported a comparable pattern in apple peel. Hydroxycinnamic acids protect the fundamental tissues from adverse UV radiation; therefore, their expanded accumulation in intense light exposure is anticipated [36]. The high UV-B exposure at higher altitudes is the key determinant for the increased synthesis of phenolic acids in plants [63]. Moreover, lower temperatures at higher altitudes also sustain secondary metabolism [33,37], particularly the accumulation of hydroxycinnamic acids. Following all the above, the Borsa habitat, characterized by a good solar exposure, low-temperature range, and brown acid soils, may explain the highest levels of hydroxycinnamic acids when compared with a moderate/partial solar irradiation and higher temperature ranges (characterizing the other two habitats).

From the flavonols group, seven phenols were identified, all were quercetin derivatives: quercetin, quercetin-rutinoside (rutin), quercetin-glucoside, quercetin-acetyl-rhamnoside, quercetin-arabinoside, quercetin-xyloside, and quercetin-diglucoside. The major flavonol identified within both species was quercetin-rutinoside (rutin), being approximately 40 times higher than most of the flavonols compounds identified, and about 2 times higher than quercetin-acetyl-rhamnoside. Among studied bilberry leaves, VMB presented the highest level (49.83 ± 0.63 mg/g plant material), where all the extracts showed a level above 40 mg/g plant material. Concerning the lingonberry leaves, again, VVIB has shown the highest amount (21.88 ± 0.19 mg/g plant material), where VVIS was half of this level. The second major flavonol compound identified in both species was quercetin-acetyl-rhamnoside with VMT presenting the highest amount (18.60 ± 0.16 mg/g plant material) in the bilberry species, and VVIB in the lingonberry species (8.01 ± 0.01 mg/g plant material). In the case of almost all flavonol compounds, the Smida location (1100 m altitude) reported the lowest values. These results suggest that a Borsa habitat-type (altitude 850 m, good solar exposure, low-temperature range, brown acid soils) may be more beneficial for the biosynthesis of major flavonols compounds, whereas a good solar exposure (low forest environment) may positively contribute to flavonols level. The existing literature [19] on bilberry leaves reported, as major flavonols compound, the quercetin glycosides, namely, quercetin-3-O-galactoside, quercetin-3-O-glucoside, quercetin hexuronides, quercetin pentosides, and a quercetin rhamnoside. Moreover, the specific quercetin-3-O-(400-(3-hydroxy-3-methyl glutaryl))-a-rhamnoside was identified in all of the morphological parts studied, being previously reported in leaves by Hokkanen et al. [23] and Ieri et al. [27]. In the study of Bujor et al. [14], investigating the lingonberry leaves, a range of 12–19% flavonols were found. There were 18 quercetin glycosides identified, whereas the quercetin-3-O-galactoside, quercetin-3-O-glucoside, quercetin rutinoside, quercetin pentosides, and quercetin-3-O-rhamnoside were in line with previous findings [16,23,25] and our study.

Concerning the anthocyanins class, the three anthocyanins identified were cyanidin-glucoside, cyanidin-arabinoside, and cyanidin-acetyl-glucoside. The anthocyanins group was present only in VMT and VMS leave extracts, but in a small amount (<0.35 mg/g). Our findings are in agreement with the study of Jaakola et al. [36], where elevated gene expression and, therefore, flavonoid biosynthesis including cyanidin glycosides owing to an increased UV exposure in bilberry leaves was described. Therefore, an assumption that anthocyanins from both bilberry leaves may have occurred in higher amounts with increased solar radiation can be made, if we consider the presence of cyanidin-arabinoside only in VMS. When compared with their related berries [64], anthocyanin synthesis is highly affected by light exposure and, as a consequence, bilberries from shaded sites [40] contained lower amounts of red pigments, as low light conditions limit photosynthetic activity. The mechanism involves firstly a decrease in carbohydrate synthesis, followed by a low level of substrate generated for secondary metabolism. Certain phenolic classes are then downregulated, with anthocyanin synthesis negatively influenced. Li et al. [62] also found that anthocyanin level as well as the flavonol content and activity of phenylalanine ammonia-lyase (PAL), and certain enzymes, were increased in the sun-exposed apple peel compared with the shaded peel, underlying the upregulation of the phenylpropanoid pathway generated by a favorable light condition. Rieger et al. [60] reported that the anthocyanins level in bilberries decreased with increasing altitude, while Roslon et al. [65] did not found a relationship between the content of anthocyanins in bilberry fruits and the position of habitats at different altitudes. In the same study, the leaves anthocyanins were not investigated as they were considered not specific for leaves.

### 3.2. Total Phenolics and Total Flavonoids

**Figure 1:**
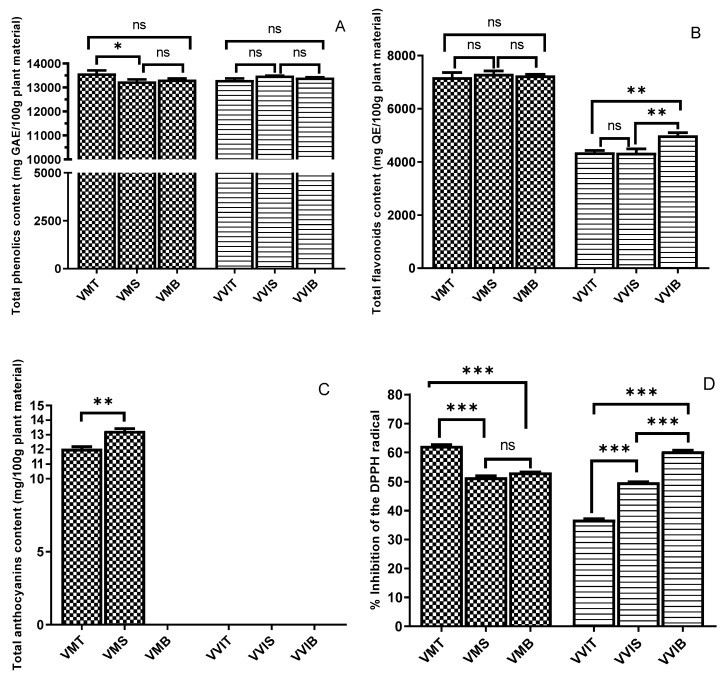
Total phenolic content (Folin–Ciocalteu method) (**A**), total flavonoids content (**B**), total anthocyanin content (**C**), and 2,2-diphenyl-1-picrylhydrazyl (DPPH) antioxidant activity (**D**) of the two species leave extracts, from all three locations. The total phenolic content of the extract is expressed as gallic acid equivalents (GAE) in mg/100 g plant material. Total flavonoid content is expressed as quercetin equivalents (QE) in mg/100 g plant material. The DPPH activity was expressed as percentage inhibition (I%). Values are reported as mean ± SD of triplicate determinations and different symbols (*, **, ***) indicate significant differences (*p* < 0.05) between the three different locations for each of the two species leave extracts, separately (one-way analysis of variance (ANOVA), multiple comparison tests, Tukey multiple range tests), while symbol (ns) indicate no significant difference. VMT, *V. myrtillus* leaves from Turda; VMS, *V. myrtillus* leaves from Smida; VMB, *V. myrtillus* leaves from Borsa; VVIT, *V. vitis-idaea* leaves from Turda; VVIS, *V. vitis-idaea* leaves from Smida; VVIB, *V. vitis-idaea* leaves from Borsa.

According to Figure 1A, the TPC among both species was very similar, whereas there were no statistical differences between the three types of lingonberry leaves. In the case of bilberry leaves, the highest level of TPC was registered by VMT with 13,588.95 ± 9.25 mg GAE/100 g plant material (135.8 ± 9.25 mg GAE/g plant material), being significantly different only from VMS, but not from VMB. In the study of Bujor et al. [19], on the same period of vegetation (September), for the bilberry leaf extracts, a TPC of 142.9 ± 19.2 (mg GAE/g dry extract) was reported, while in the most recent study on lingonberry of the same author [14], the lingonberry leaves extract shown a TPC of 158.9 ± 6.0 (mg GAE/g dry extract). These results are in the same range as our findings. Nevertheless, the *Vaccinium* plants have the same Romanian origin, but different habitats and environmental factors. In the study of Tian et al. [59], the lingonberry leaf ethanolic extracts showed a TPC of 859.5 ± 9.9 (GAE mg/100 mL), while for bilberry leaf ethanolic extracts, a TPC of 201.7 ± 18.2 (GAE mg/100 mL) was found. A possible explanation for the evident differences in contrast to our results may lie in the geographical location, Finnish versus Romanian. According to the same study [59], a higher value of Folin–Ciocalteau was found in the extracts from leaves than in the extract from berries and branches, and the leaf extracts showed higher antioxidative activities (3–20-fold in ORAC assay, 10–20 fold in TRAP) than the berry extracts, in association with the higher contents of phenolic compounds in the leaf extracts [59]. However, regarding our findings, it may be stated that the different habitats did not statistically influence the TPC of both bilberry and lingonberry leaves, considering the high fluctuation in amounts in the different phenolic sub-classes among the three different locations.

Figure 1B presents the TFC of the leaves extracts from both examined species. Among the studied bilberry leaves, there was no statistical difference with the habitat variation, with all three extracts having similar levels of approximately 7300 mg QE/100 plant material. The bilberry species had twofold higher TFC when compared with lingonberry. This may be explained by the fact that chromatographic profiles of flavonoids, but not only them, are different among bilberry, blueberry, lingonberry, and cranberry [66,67]. In the case of lingonberry extracts, the VVIB registered the highest value (4994.18 ± 8.03 mg QE/100 plant material), being statistically different from the other two habitats. Uleberg et al. [37] found that the amount of flavan-3-ols was higher in bilberries growing at lower temperatures, a fact that might explain why Borsa habitat, with lower temperatures (ranging between 13 °C and 8 °C), registered higher amounts. In the study of Mikulic-Petkovsek et al. [40], low levels of flavanols were found in fruits collected in shaded forests characterized by a low photosynthetic active radiation (PAR), and high flavanol amounts in bilberries from sun-exposed locations with high PAR. Jaakola et al. [36] reported that the levels of flavan-3-ols were significantly higher in bilberry leaves exposed to direct sunlight. Considering that Borsa collection place had a good solar exposure when compared with partial or moderate exposure in the other two habitats, these previous findings might explain our results.

Other articles [60,63] reported that increased solar exposure by higher altitudes contributes directly to increased flavonoids content in plants, which is partially true in our study considering that Borsa location (850 m altitude) had the highest level, and Smida location (1100 m) did not. Moreover, another study investigating the bilberry leaves concluded that leaves collected from open and forest areas showed that synthesis and accumulation of flavonoids were delayed in the forest compared with the high light open sites [24]. Several flavonoids and hydroxycinnamic acids act as characteristic UV shields and contribute to the plants’ protective mechanism determined by high irradiation sites [60].

### 3.3. Total Anthocyanin Content

To our knowledge, this is the first study investigating the variation of the amounts of anthocyanins in wild-grown bilberries and lingonberries leaves in correlation to the geographical habitat. According to Figure 1C, only bilberry leaves via VMT and VMS extracts were found to have anthocyanin content. The anthocyanin profile contained small amounts of cyanidin glycosides, whereas VMS registered the highest level, precisely 13.29 ± 0.13 mg/100 g plant material. Our results matched well with the results of a previous comparison of eastern and southern European plants with those from Scandinavia [68], suggesting that a higher altitude may provide an increased sunlight exposure, and thus a higher anthocyanins content. The low levels of total anthocyanin content might be owing to a non-specificity in the leaves of berries when compared with the fruits.

### 3.4. DPPH Antioxidant Activity

The percentage inhibition of the DPPH radicals for tested leaf extracts is shown in Figure 1D. For bilberry leaves, VMT showed the best radical scavenging capacity based on the DPPH assay expressed as percentage inhibition of DPPH radicals (also as Trolox equivalents, precisely 310.74 mMT/100 g), while in the case of lingonberry leaves, the VVIB had the highest percentage inhibition of the DPPH radicals (and as Trolox equivalents, precisely 320.83 mMT/100 g). These results could be explained by the highest value registered for TPC, in the case of bilberry leaves, and considering the well-known correlation between increased phenolic content and strong antioxidant capacity, whereas for VVIB, the highest TFC value reported may be responsible for the scavenging capacity, as previously reported in the literature [69]. Previous articles have reported that more solar exposure at increased altitude contributed to elevated biosynthesis of ortho-dihydroxylated flavonoids [34,63], as well as a better radical scavenger capacity [70]. In the case of lingonberry leaves, flavanols, flavonols, and caffeic acid derivatives bring highly antioxidant 1,2-dihydroxyphenyl moieties, whereas coumaric acid derivatives display the less antioxidant monohydroxyphenyl moiety. For similar phenolic contents in the case of lingonberry leaves, the *V. vitis-idaea* leaves from Turda (VVIT) present significantly lower antioxidant capacity than both VVIS (−26%) and VVIB (−39%) (Figure 1D). Concerning the higher levels of flavanols, and precisely of procyanidin dimers and procyanidin trimer, compared with VVIT, as well as for VVIB, a significant contribution via feruloylquinic acid might explain the differences. Extension and terminal epicatechin units in flavanols were already proven to be similarly reactive in the quenching of the nitrogen-centered DPPH radical [71]. In the study of Tian et al. [59], the DPPH radical scavenging capacity of the berry leaf extracts varied among species and cultivars, whereas within 10 min, all the leaf extracts succeeded to capture around 80% of DPPH radicals.

The difference in reactivity of leaf extracts, from the three different habitats, in the DPPH test can be attributed to their varying contents in polyphenols containing dihydroxyphenyl moieties, molecular sizes, like for flavanols [14], or the presence of unidentified antioxidant substances. Soobrattee et al. [72] classified the antioxidant activity in the following order: procyanidin dimer > flavan-3-ols > flavonols > hydroxycinnamic acids > simple phenolic acids. Heim et al. [73] explained why the proanthocyanidins (procyanidin dimers and procyanidin trimer) as oligomers and polymers of flavan-3-ols exhibit stronger DPPH capacity, namely owing to more catechol groups, coupled with C3-OH and C4-C8 linkage.

### 3.5. Assessment of Antimicrobial Capacity

The studied leaves extract registered antibacterial and antifungal capacity towards bacteria and fungal strains. The results of MIC are described in Table 2, and those of MBC are provided in Table 3, for both bacteria and fungi strains. An important range of bacteriostatic effects of the bilberry and lingonberry leaves extracts was reported, depending on the tested strain.

In the case of bilberry leaves, towards *S. aureus*, the best antibacterial activity was registered for both VMT and VMB (MIC = 0.06 and MBC = 0.12 mg/mL). This result may be because of the increased TPC, considering that several studies underlined the fact that polyphenols may attack an important number of bacteria, and the antimicrobial capacity depends on interactions between polyphenols and bacterial cell surface [74,75]. *R. equi* was the most sensitive strain towards all the bilberry extracts, whereas *E. faecalis* Gram-positive strain was the most resistant one.

Towards the lingonberry extracts, *S. aureus* and *E. faecalis* exhibited a higher resistance in comparison with *R. equi.* Considering the Gram-negative strains, *E. coli enterotoxigen* was not as sensitive as *K. pneumonia* and *P. aeruginosa* towards extracts’ antibacterial effects. In the case of lingonberry species, the same antibacterial pattern was registered as for Gram-positive ones.

The highest inhibitory activity among bilberry species against all the strains is attributed to the VMT and VMB, while for lingonberry, it seems that natural habitat conditions did not influence the antibacterial effect, but the antifungal effect, only in the case of *Candida zeylanoides*. It can be concluded that the Gram-positive strains were much more sensitive to all the tested extracts when compared with the Gram-negative ones.

There are only a few studies reporting the antibacterial capacity of bilberry and lingonberry species. In vitro antimicrobial effect of flavonol glycosides, anthocyanins, procyanidins, and flavan-3-ols groups derived from lingonberry juice were demonstrated towards *S. mutans* and *F. nucleatum* [76]. Moreover, the antibacterial capacity of fruits and leaves of bilberry in different types of solvents, like water, ethanol, and ethyl acetate, was tested on *E. coli*, *E. faecalis*, and *P. vulgaris*, and it was reported that all extracts had a higher effect towards *E. faecalis* and *P. vulgaris* [77]. The study of Tian et al. [59] demonstrated that the extracts of lingonberry leaves, hawthorn leaves, sea buckthorn leaves, Saskatoon leaves, and raspberry leaves registered high inhibitory effects towards *S. aureus*, *L. monocytogenes*, and *B. cereus*. The findings also suggested increased sensitivity of Gram-positive in contrast to Gram-negative bacteria to the phenolic extracts. Moreover, the same study found that the TPC and the content of non-flavonoid phenolics presented a stronger correlation with the inhibitory effects on *S. aureus* and *Bacillus cereus* when compared with TFC. Another study [78] established that lingonberry fruit extracts containing mainly type-A proanthocyanidins may be bactericidal against *S. aureus* or inhibit the hemagglutination of *E. coli.* Considering that both species had important amounts of proanthocyanidins (procyanidin dimer II, procyanidin trimer), this hypothesis could explain the antibacterial effects on *S. aureus* and *E. coli*. Several mechanisms of action in the growth inhibition of bacteria are involved, such as destabilization of the cytoplasmic membrane, permeabilization of the plasma membrane, inhibition of extracellular microbial enzymes, direct actions on microbial metabolism, and deprivation of the substrates required for microbial growth [79].

Regarding the antifungal capacity, both species, in the case of all three types of habitats, had the same effect towards *Candida albicans*, precisely none (MIC = 125 and MFC = 250 mg/mL) when compared with control Fluconazole (MIC = 15.62 and MFC = 31.25 mg/mL), with the highest antifungal effect towards *Candida parapsilosis* (MIC = 31.25 and MFC = 62.5). Against *Candida zeylanoides,* the highest inhibitory potential was registered by VMB and VMT (MIC = 31.25 and MFC = 62.5 mg/mL), as well as VVIS, respectively. A possible explanation of why *Candida* species were not sensitive to our berry leaf extracts could be explained by the lack of ellagitannins, reported previously as the main antimicrobial compounds against these microorganisms [80].

### 3.6. Assessment of Antimutagenic Effects of Bilberry and Lingonberry Leaves

The investigation and discovery of antimutagenic properties of plants are of great practical and therapeutic importance in pharmacology and medicine. Research over the past few years has revealed that mutation has a key role in carcinogenesis [81]. The wild bilberry and lingonberry leaf extracts were tested for their antimutagenic activity, considering our recent review study on their significant antioxidant capacity [1]. The influence of the habitat conditions, as well as the type of *Vaccinium* species, had an important influence on the number of revertants in *S. typhimurium* TA98 and TA100. The antimutagenicity potential of both species towards *S. typhimurium* TA98 and TA100 is reported in Table 4, whereas the tested direct-acting mutagens were 4-NPD for TA98 and sodium azide (NaN3) for TA 100.

Concerning *S. typhimurium* TA98, the leaf extracts proved to significantly inhibit the number of revertants of strain TA98 induced by 4-NPD. Therefore, moderate antimutagenic activity was reported for both types of species, whereas the higher inhibition was registered by the bilberry leaves, precisely VMT (31.95%), closely followed by VMS (31.44%). A possible explanation for this could be the fact that the VMT sample had an increased scavenging capacity, as the literature constantly links the antioxidant potential with the antimutagenic capacity of different types of plant extracts [82,83]. Moderate inhibition of around 25% was registered by all the lingonberry types of leaves, a fact that might be explained by the lack of statistically significant levels of TPC among the three extracts.

Towards TA100, both types of species registered a higher antimutagenic activity, suggesting that *S. typhimurium* TA100 was much more sensitive to *Vaccinium*-type of leaf extracts. Following Table 4, all three types of bilberry leaves showed a strong inhibition capacity (>40%), whereas the best inhibition was exhibited by the VMT (43.26%) extract, closely followed by VMS and VMB. Regarding the lingonberry leaves, all of the extracts proved a moderate antimutagenic effect, whereas the VVIT extract inhibited the mutagenic effect of sodium azide of more than 36%, while the VVIS had the lowest inhibition percentage (29.79%). The best antimutagenic capacity was registered toward the strain *S. typhimurium* TA100 by all the leaf extracts.

In the case of both species, all the above-mentioned favorable effects are more likely to be associated with a high content of flavanols and flavonols, which significantly decrease the mutagenic activity of the standard mutagens examined. To the best of our knowledge, this is the first study evaluating the antimutagenic activity of Nord-West Romanian wild bilberry and lingonberry leaves, thus with significant novelty for the present paper. Moreover, the literature lacks in studies investigating this specific features of bilberry and lingonberry fruits or leaves. In the study of Smith et al. [84], the antimutagenic activity of different berry extracts was investigated. Among the tested berries were strawberry, raspberry, and blueberry of different fresh cultivars, and in several kinds of solvents (H_2_O, ethanol, methanol). The antimutagenic inhibition range was between 23% and 53%. Another study [85] investigating the capacity to prevent mutation induced by two promutagenic dietary quinolines, namely MeIQ and 4-NQO of the *V. floribundum* and *V. myrtillus* berries extracts, reported being inactive at concentrations up to 1000 g/plate. In the recent review study on flavonoids’ bioactivity [86], it was reported that plant flavonoids exhibit an important antimutagenic activity. Moreover, cranberry (*V. macrocarpon*), as a significant source of polyphenols, has been reported within vitro antimutagenic properties [2,87].

Therefore, regarding the existing data, we can state that the berries contain anthocyanins and procyanidins, constantly reported for their strong antioxidative activity, leading to both in vitro and in vivo antibacterial and antimutagenic activities [88]. In this study, the hydroethanolic extracts from leaves of *V. myrtillus* L. and *V. vitis-idaea* L. did inhibit the mutations on the Ames *Salmonella* test. Besides, the present study has shown for the first time that hydroethanolic extracts from leaves of wild bilberry and lingonberry are a promising source for its antimutagenic compounds. These results indicate that it may be considered to be a safe and useful agent for the prevention of mutations.

## 4. Conclusions

The influence of natural habitats on the level of individual phenolic compounds and biological activities were examined, and considerable variations in phenolic profile and significant differences of bioactivities between bilberry and lingonberry leaves, collected from three different locations, were observed.

This study reports a qualitative analysis of bilberry and lingonberry leaves with structures proposed for 21 phenolic compounds. Quantitative analysis revealed that flavonoids class contribute more than half of the phenolic pool in leaves; whereas for bilberry species, rutin represents 50% of this subclass; and for lingonberry species, the flavanols comprise the majority via gallocatechin, epigallocatechin, catechin, and procyanidin trimer. Of significant novelty was the antimutagenic testing among these species, at different habitats, concluding that bilberry leaves have a stronger antimutagenic capacity, whereas better sun exposure may contribute to an increased flavonols synthesis, leading to better antioxidant and antimutagenic activities. Regarding the antimicrobial effects of the studies species, the Gram-positive bacteria were more susceptible to the activity of the extract, presenting high antibacterial effects, whereas the antifungal capacity was low. 

Thus, the hypothesis that plants from higher altitudes contain higher amounts of radical scavenging compounds as a result of their exposure to more severe climatic conditions including enhanced solar radiation cannot be affirmed in general. On the basis of our results, distinct differences between the amounts of phenolic compounds due to habitat-derived conditions (altitude, solar exposure, temperature range, and so on) can be expected at least in the case of the flavanols, flavonols, hydroxycinnamic acids, and anthocyanins investigated in these species.

## Figures and Tables

**Table 1:** The phenolic compounds content in the leaves of bilberry and lingonberry using HPLC-DAD-ESI-MS and expressed as mg/g.

Phenolic Compounds		Retention Time R_t_ (min)	UV λ_max_ (nm)	[M+H]^+^ (m/z)	VMT	VMS	VMB	VVIT	VVIS	VVIB
**Flavanols**	**Gallocatechin**	2.97	279	307, 290	7.59 ± 0.07 ^b^	15.37 ± 0.14 ^a^	4.84 ± 0.05 ^c^	35.10 ± 0.24 ^b^	46.81 ± 0.38 ^a^	31.41 ± 0.21 ^c^
	**Epigallocatechin**	4.24	279	307, 290	n.d	6.56 ± 0.06	n.d	25.24 ± 0.72 ^b^	35.97 ± 0.23 ^a^	23.35 ± 0.61 ^c^
	**Catechin**	12.58	280	291	9.87 ± 0.07 ^a^	4.79 ± 0.07 ^c^	5.38 ± 0.09 ^b^	18.51 ± 0.21 ^b^	21.57 ± 0.2 ^a^	17.43 ± 0.18 ^b^
	**Epicatechin**	13.11	280	291	4.31 ± 0.03 ^b^	9.66 ± 0.08 ^a^	n.d	n.d	n.d	2.78 ± 0.03
	**Procyanidin dimer I**	11.33	280	579, 291	n.d	n.d	n.d	6.38 ± 0.05 ^b^	8.36 ± 0.06 ^a^	6.27 ± 0.03 ^b^
	**Procyanidin dimer II**	19.74	280	579, 291	12.68 ± 0.11 ^a^	12.13 ± 0.12 ^a^	8.70 ± 0.07 ^b^	2.20 ± 0.05 ^b^	4.23 ± 0.04 ^a^	4.61 ± 0.03 ^a^
	**Procyanidin trimer**	13.89	280	865, 291	21.84 ± 0.21 ^b^	24.30 ± 0.72 ^a^	10.09 ± 0.12 ^c^	12.92±0.12 ^c^	14.21±0.16 ^b^	18.84±0.22 ^a^
**Hydroxycinnamic acids**	**Chlorogenic acid**	12.01	281, 329	355, 163	3.34 ± 0.03 ^c^	3.85 ± 0.02 ^b^	5.94 ± 0.05 ^a^	0.79 ± 0.01 ^b^	n.d	1.16 ± 0.01 ^a^
	**Feruloylquinic acid**	14.79	283, 330	369	55.37 ± 0.42 ^b^	47.66 ± 0.39 ^c^	59.65 ± 0.44 ^a^	31.05 ± 0.18 ^b^	24.61 ± 0.24 ^c^	33.42 ± 0.37 ^a^
	**Caffeoylarbutin**	17.20	288, 330	435	n.d	n.d	n.d	6.45 ± 0.04 ^a^	3.42 ± 0.02 ^c^	5.14 ± 0.05 ^b^
	**Dicaffeoylquinic acid**	20.08	282, 329	517, 163	5.01 ± 0.05 ^a^	4.05 ± 0.04 ^b^	n.d	1.77 ± 0.02 ^a^	0.93 ± 0.01 ^b^	1.74 ± 0.01 ^a^
**Flavonols (quercetin derivatives)**	**Quercetin-rutinoside (Rutin)**	15.35	263, 355	611, 303	44.91 ± 0.21 ^b^	42.34 ± 0.19 ^c^	49.83 ± 0.63 ^a^	18.61 ± 0.19 ^b^	11.45 ± 0.10 ^c^	21.88 ± 0.19 ^a^
	**Quercetin-glucoside**	16.20	263, 355	465, 303	1.42 ± 0.01 ^b^	1.29 ± 0.01 ^c^	2.37 ± 0.02 ^a^	3.05 ± 0.03 ^a^	2.23 ± 0.03 ^b^	1.91 ± 0.02 ^c^
	**Quercetin-acetyl-rhamnoside**	17.83	263, 356	493, 303	18.60 ± 0.16 ^a^	12.67 ± 0.10 ^c^	15.47 ± 0.14 ^b^	6.10 ± 0.04 ^b^	1.71 ± 0.01 ^c^	8.01 ± 0.07 ^a^
	**Quercetin-arabinoside**	18.69	262, 355	435, 303	1.55 ± 0.01 ^a^	1.53 ± 0.01 ^a^	1.39 ± 0.01 ^b^	0.41 ± 0.01 ^b^	0.07 ± 0.01 ^c^	0.61 ± 0.01 ^a^
	**Quercetin-xyloside**	18.98	262, 355	435, 303	1.47 ± 0.01 ^b^	1.30 ± 0.01 ^c^	1.53 ± 0.01 ^a^	0.45 ± 0.01 ^b^	0.05 ± 0.01 ^c^	0.62 ± 0.01 ^a^
	**Quercetin-diglucoside**	21.15	263, 355	628, 303	0.91 ± 0.01 ^c^	1.42 ± 0.01 ^a^	0.17 ± 0.01 ^b^	3.11 ± 0.03 ^b^	3.93 ± 0.05 ^a^	1.12 ± 0.01 ^c^
	**Quercetin**	21.88	261, 355	303	3.69 ± 0.03 ^a^	3.26 ± 0.04 ^b^	1.16 ± 0.06 ^c^	4.78 ± 0.04 ^a^	3.31 ± 0.02 ^b^	2.61 ± 0.02 ^c^
**Anthocyanins**	**Cyanidin-glucoside**	11.02	210, 517	449, 287	0.28 ± 0.01 ^a^	0.29 ± 0.01 ^a^	n.d	n.d	n.d	n.d
	**Cyanidin-arabinoside**	11.78	214, 517	419, 287	n.d	0.30 ± 0.01	n.d	n.d	n.d	n.d
	**Cyanidin-acetyl-glucoside**	14.28	218, 518	491, 287	0.33 ± 0.01 ^a^	0.29 ± 0.01 ^b^	n.d	n.d	n.d	n.d

Values (expressed as mean values ± SD, mg/g, *n* = 3) in the same row followed by different letters (a–c) indicate significant differences (*p* < 0.05) between the three different locations, individual for each type of species (one-way analysis of variance (ANOVA); multiple comparison test; Tukey multiple range test (*p* = 0.05); GraphPad Prism Version 8.0.1, Graph Pad Software, Inc., San Diego, CA, USA). VMT, *V. myrtillus* leaves from Turda; VMS, *V. myrtillus* leaves from Smida; VMB, *V. myrtillus* leaves from Borsa; VVIT, *V. vitis-idaea* leaves from Turda; VVIS, *V. vitis-idaea* leaves from Smida; VVIB, *V. vitis-idaea* leaves from Borsa; n.d, not detected.

**Table 2:** Minimum inhibitory concentration (MIC) of bilberry and lingonberry leaves expressed as mg/mL.

Type of Strains	Gram-Positive			Gram-Negative			Fungi		
Sample	*S. aureus*	*E.* *fecalis*	*R. equi*	*E. coli enterotoxigen*	*K. pneumonia*	*P. aeruginosa*	*Candida* *albicans*	*Candida* *zeylanoides*	*Candida* *parapsilosis*
mg/mL									
VMT	0.06	0.12	0.06	0.24	0.24	0.24	125	31.25	31.25
VMS	0.12	0.24	0.06	0.48	0.12	0.24	125	62.5	31.25
VMB	0.06	0.12	0.06	0.48	0.12	0.24	125	31.25	31.25
VVIT	0.12	0.12	0.06	0.48	0.12	0.96	125	62.5	31.25
VVIS	0.12	0.12	0.06	0.48	0.12	0.96	125	31.25	31.25
VVIB	0.12	0.12	0.06	0.48	0.12	0.96	125	62.5	31.25
Fluconazole μg/mL	-			-	-	-	15.62	7.81	15.62
Streptomicyn μg/mL	0.03	0.06	0.06	0.12	0.06	0.06			

**Table 3:** Minimum bactericidal/fungicidal concentration (MBC/MFC) of bilberry and lingonberry leaves expressed as mg/mL.

Type of Strains	Gram-Positive			Gram-Negative			Fungi		
Sample	*S. aureus*	*E.* *fecalis*	*R. equi*	*E. coli enterotoxigen*	*K. pneumonia*	*P. aeruginosa*	*Candida* *albicans*	*Candida* *zeylanoides*	*Candida* *parapsilosis*
mg/mL									
VMT	0.12	0.24	0.12	0.48	0.48	0.48	250	62.5	62.5
VMS	0.24	0.48	0.12	0.96	0.24	0.48	250	125	62.5
VMB	0.12	0.24	0.12	0.96	0.24	0.48	250	62.5	62.5
VVIT	0.24	0.24	0.12	0.96	0.24	1.92	250	125	62.5
VVIS	0.24	0.24	0.12	0.96	0.24	1.92	250	62.5	62.5
VVIB	0.24	0.24	0.12	0.96	0.24	1.92	250	125	62.5
Fluconazole μg/mL	-			-	-	-	31.24	15.62	31.24
Streptomicyn μg/mL	0.06	0.12	0.12	0.24	0.12	0.12			

**Table 4:** Antimutagenicity capacity towards *Salmonella typhimurium* TA98 and TA100 strains.

Samples	Number of Revertants			
	TA 98		TA100	
	Mean ± S.D	Inhibition %	Mean ± S.D	Inhibition %
Negative Control	9.25 ± 3.6 ^a^		9.25 ± 2.4 ^a^	
VMT	132 ± 3.2	31.95	198 ± 4.2	43.26
VMS	133 ± 4.4	31.44	201 ± 6.3	42.4
VMB	137 ± 3.6	29.38	202 ± 5.4	42.12
VVIT	144 ± 4.7	25.77	223 ± 2.6	36.1
VVIS	145 ± 2.1	25.25	245 ± 4.3	29.79
VVIB	144 ± 5.9	25.77	234 ± 7.9	32.95
4-NPD ^b^	194 ± 3.3	-	-	-
NaN_3_^b^	-	-	349 ± 15.22	-

^a^ Values expressed are means ± S.D of three replications. ^b^ 4-nitro-ophenylenediamine (4-NPD) and NaN_3_ were used as positive controls for *Salmonella thyphimurium* TA98 and TA100 strains, respectively.
